# Association study between polymorphisms in *MIA3*, *SELE*, *SMAD3* and *CETP* genes and coronary artery disease in an Iranian population

**DOI:** 10.1186/s12872-022-02695-6

**Published:** 2022-06-30

**Authors:** Sima Rayat, Nasim Ramezanidoraki, Nima Kazemi, Mohammad H. Modarressi, Masoumeh Falah, Safoura Zardadi, Saeid Morovvati

**Affiliations:** 1 Department of Biology, School of Basic Sciences, Science and Research Branch, Islamic Azad University, Tehran, Iran; 2 Department of Medical Genetics, Tehran University of Medical Sciences, Keshavarz Blvd, Tehran, Iran; 3 Department of Genetics, Faculty of Advanced Sciences and Technology, Tehran Medical Sciences, Islamic Azad University, Tehran, Iran; 4 ENT and Head and Neck Research Center, The Five Senses Health Institute, School of Medicine, Iran University of Medical Sciences, Tehran, Iran

**Keywords:** *MIA3*, *SELE*, *SMAD3*, *CETP*, CAD, Association, Polymorphism

## Abstract

**Background:**

Coronary artery disease (CAD) is the most common heart disease. Several studies have shown association between some polymorphism in different genes with CAD. Finding this association can be used in order to early diagnosis and prevention of CAD.

**Method:**

101 CAD patients with ≥ 50% luminal stenosis of any coronary vessel as case group and 111 healthy individuals as control group were selected. the polymorphisms were evaluated by ARMS-PCR and RFLP-PCR methods.

**Result:**

The results of this study show that there is no significant association between rs17228212, rs17465637, and rs708272 and risk of CAD. But there is significant association between risk of CAD and rs5355 (*p*-value = 0.022) and rs3917406 (*p*-value = 0.006) in total cases, and rs5882 (*p*-value = 0.001) in male cases.

**Conclusions:**

Our findings revealed a significant interaction between *CETP* SNPs and *CETP* activity for affecting HDL-C levels. The *SELE* gene is a known cell adhesion molecule with a significant role in inflammation. Studies about possible linkage between *SELE* gene polymorphisms and the development of CAD are conflicting. We have found a significant association between polymorphisms of *SELE* gene and risk of CAD.

## Background

Cardiovascular diseases are among the major causes of fatality worldwide. Coronary artery disease (CAD) is a cardiac disorder with the highest prevalence resulting from coronary artery stenosis and atheroma plaque development. CAD is caused by multiple factors as a result of genetic parameters and environmental impacts and their interplays. Traditionally, several risk factors can merely elucidate a limited number of cases found in reports [1]. Thus, genetic parameters play a critical role in disease development. Studies have focused on multiple CAD-related genes. The hypothesis that CAD results from the heredity of genes with high or low penetrance responsible for inflammation procedures are nowadays of great interest [2].

In a genome-wide association investigation [3], specific single nucleotide polymorphism (SNP) at chromosome 1q41, rs17465637, was reported to have an association with CAD and myocardial infarction (MI). The SNP exists in the melanoma inhibitory activity-3 gene (*MIA3*), also referred to as TANGO, ARNT, D320, ODCD2, TANGO1, and UNQ6077 with a wide-ranging expression pattern. Existing evidence shows that TANGO is a member of a gene family of four homologous proteins, TANGO, MIA, OTOR (FDP, MIAL), and MIA2. By joining TANGO with the homologous proteins, a gene family is defined that shares essential structural specifications, substantial homology at both nucleotide and protein levels, and analogous arrangement in the genome [4]. A 14-kDa protein with unidentified activity hitherto is encoded by the *TANGO* gene. The down-regulation or loss of *TANGO* was reported in all hepatocellular and colon cell lines in comparison to primary human hepatocytes or normal colon epithelial cells in respective order and in the majority of the tumor samples including malignant melanoma and colon and hepatocellular carcinoma in comparison to tumor-free tissues. Therefore, *TANGO* was proposed to possibly serve as a tumor-suppressive gene in malignant melanoma [5, 6]. Moreover, *TANGO* can supposedly influence an increase in the risk of CAD via inflammatory procedures. The critical function of the inflammatory modes of action in forming atheroma is supported by copious data. This process involves leukocyte mobilization and pivotal participation of proinflammatory cytokines in the primary phase of atherogenesis [7]. Endothelial cells are activated for inflammation, followed by the entry of monocytes and other leukocytes into the atheroma. The direct binding of TANGO protein occurs to the leukocyte-specific b2-integrin CD11c/CD18, being responsible for leukocyte adherent interplays with vascular endothelium. Experimentally, it is evidenced that the TANGO protein modulates CD11c/CD18 activity, thereby reducing the adhesion and promoting monocyte migration through the endothelium [8]. After trans-migration, monocytes are differentiated into macrophages, forming the fatty streak that primarily indicates atherosclerosis in the arterial intima. Thus, atherosclerosis is driven by inflammation, eventually triggering thrombotic plaque consequences, often causing myocardial infarction (MI) and CAD [7]. In some earlier reports, the rs17465637 and CAD were also reported to have an association [9].

As stated above, atherosclerosis develops and progresses by the mobilization and coupling of circulatory leukocytes to inflammation sites inside the vascular endothelium [10]. This progression is mainly under the modulation and regulation of wide-ranging adherence molecules, such as selectins, integrins, immunoglobins, and chemokines [11]. The selectins are a class of cell adherence molecules responsible for chronic and acute inflammation procedures. The expression of these single-chain transmembrane glycoproteins occurs on endothelial cells when they are stimulated by inflammatory cytokines. The adherence of pathological cells to endothelium is determined by the expression profile of selectins on the vascular wall because the adherence is facilitated when endothelial selectins are present. A connection between selectins and the incidence of CAD is supported by growing evidence [12]. The *SELE* gene is situated on chromosome 1q22-q25. P-selectin, E-selectin, and L-selectin form the family of selectins. These three selectins differ fundamentally in their distribution, activation, and expression. The E-selectin expression only happens on the actuated endothelium and mobilizes polymorphonuclear leukocytes, myeloid cells, and T-lymphocytes to the inflammation areas [13]. The P-selectin expression occurs on both the endothelial cells and platelets and has an instant migration to the plasma membrane after exposure to some free radicals, nitric oxide synthase suppressors, and other intermediaries [14]. The expression of L selectin is seen on most kinds of leukocytes, as well as on some neutrophils and myeloid cells [13]; it acts as a homing receptor for lymphocytes to peripheral lymph nodes. Similar to E-selectin, L-selectin mobilizes cells to inflammation lesions. Currently, the *SELE* gene is reported as a high-risk factor involved in atherosclerosis development. An association between variations in rs3917406 and rs5355 (G1839T) of the *SELE* gene with CAD was reported previously [15, 16].

The present study focused on the *SMAD3* gene that plays a role in the TGFB signaling route and has a major contribution to the inflammation procedure. Eight Smad family members (Mad-homologues (MADH), *MADH2*, *MADH4*, and *MADH7* map to chromosome 18q21-22, a tumor suppressor locus, *MADH3* and *MADH6* map to chromosome 15q21-22, and *MADH5*, *MADH1*, and *MADH8* to chromosomes 15q31, 4 and 13, respectively) are encoded by the human genome [17]. Three functional subfamilies of Smads include receptor-activated Smads (R-Smads: Smad1, Smad2, Smad3, Smad5, and Smad8), the phosphorylation of which is done by type I receptors; common mediatory Smads (Co-Smads: Smad4), which oligomerizes with activated R-Smads; and inhibitive Smads (I-Smads: Smad6 and Smad7), which are stimulated by members of the TGF-β family. The last one applies a negative feedback impact via competition with R-Smads for receptor interplay and through labeling the receptors for disintegration [18]. TGF-β1/SMAD3-dependent routes have a major mediatory contribution to diverse biological impacts of TGF-β1, including cell multiplication, immunity repression, and inflammation [19]. A heteromeric complex with the type II receptor is formed by binding TGF-β1 to its type I receptors, which then phosphorylates and activates the TGF-β1 downstream signaling molecules SMAD2 and SMAD3. After the heterodimerization of activated SMAD2 or SMAD3 with SMAD4, the mentioned complex is introduced into the nucleus, wherein it is engaged in regulating its target gene expression [19, 20]. Marked activation of myocardial TGF-β1 expression occurs in the cardiovascular system of patients with hypertrophic or dilated cardiomyopathy and in experimental models of myocardial hypertrophy and MI [21]. In other investigations, TGF-β signaling is proposed to be critical for suppressing the expression of inflammatory genes in healing infarcts by mediating an inflammatory infiltrate [22]. TGF-β1 is critical in the pathogenicity of infarct healing, cardiac remodeling. Through a cascade of intricate reactions, TGF-β modulates the balance of pro-inflammatory and anti-inflammatory T-cells in the immune system. SMAD3 has a major contribution to the down-regulation of T-cells and the rise of FoxP3 expression, a key stage for the differentiation of regulative T-cells [23]. The imbalance of proinflammatory Th17 and regulative T-cells is reported in acute coronary syndrome [24]. In general, few studies are available clinically on TGF-β1/SMAD3 signals in sizable groups of patients. In cardiovascular physiological processes, another regulative system is the lipoprotein system, where the anti-atherosclerotic impacts of HDL are practiced via a route called reverse cholesterol transfer (RCT), which is a cholesterol transportation route from extrahepatic cells and tissues to the liver [25]. This process involves the removal of cholesterol and phospholipids from the cell by the adenosine triphosphate-dependent transporter (ABCA1) to A-I apolipoproteins to form non-esterified HDL. Eventually, cholesterol is esterified by the plasma enzyme lecithin-cholesterol-acyltransferase (LCAT) to form mature HDL. This cholesterol is then taken directly or indirectly to the liver to be excreted in the bile. Indirectly, esterified cholesterol is transported to VLDL / LDL lipoproteins by the action of cholesteryl ester transfer protein (CETP) and finally to endocytosis by liver cells. In the direct pathway, HDL binds to liver receptors (SR-BI) and fat enters the liver cells [26]. This study focused on the main *CETP* gene in the RCT procedure. The *CETP* gene is located on 16q13, encoding a hydrophobic glycoprotein. Via participation in the RCT route, the cholesterol homeostasis in the body is maintained by cholesteryl ester transfer protein (CETP) [27].

CETP protein is a key plasma glycoprotein that is primarily produced by the liver and affects circulating HDL levels by transporting esterified cholesterol from HDL to APOB-containing particles instead of receiving triglycerides. As a result of this transfer, APOB-containing particles that are atherogenic increase and HDL levels decrease [28]. There are reports of declined levels of high-density lipoprotein cholesterol (HDL-C) and raised risk of CAD caused by the elevated activity of the *CETP* gene in some studies [29]. The influences of the two polymorphisms, rs708272 (G277A) and rs5882 (I405V), and the risk of vascular diseases has also been demonstrated previously [30]. According to existing reports, multiple genetic variants in the *CETP* gene have associations with changes in the plasma concentrations and function of CETP, HDL-C plasma concentrations, LDL and HDL particle size, and possibly the risk of CAD. This research aims to investigate the relationship between rs17465637 in the *MIA3* gene, rs3917406 and rs5355 in the *SELE* gene, rs17228212 in the *SMAD3* gene, and rs708272 and rs5882 in the *CETP* gene, with the risk of CAD.

## Materials and methods

### Ethics statement

After the approval of this study by the Ethics Committee of Rasad Pathobiology and Genetic Laboratory (Tehran, Iran), all participants presented written informed consent forms before participating in this research.

### Subject selection

From April 2015 to March 2016, two Iranian groups were enrolled at Baqiyatallah Hospital, Tehran. The research sample consisted of 101 case-patients and 111 age- and sex-frequency-matched controls. CAD populations with either (a) the presence of stenosis ≥ 50% in a minimum of one main segment of coronary arteries (the right coronary artery, left circumflex, or left anterior descending arteries) by coronary angiography, (b) symptoms representing angina pectoris, electrocardiographic changes, and elevations of cardiac enzymes based on the criteria of the World Health Organization, and (c) a certified record of coronary artery bypass graft or percutaneous coronary intervention were included in the study. Exclusion criteria were patients with congenital heart disease, cardiomyopathy, and valvular disease. The control groups resided in the same populations as the cases and were specified to be without CAD, cardiovascular and cerebrovascular diseases, and peripheral atherosclerotic arterial disease by medical records, examining clinically, and electrocardiography. Participants with acute liver and/or kidney disease were also subjected to exclusion. Data on demographic variables, medical records, using a medication, and lifestyle factors were collected using structured questionnaires by skilled interviewers. The participants were described as smokers and nonsmokers. Subjects with a smoking frequency of below 100 cigarettes in the previous lifespan were grouped as nonsmokers; otherwise, they were determined as smokers. Body mass index (BMI) was obtained via dividing the weight (kg) by the square of the height (m). Additionally, patients with hypertension were considered as those having a previous diagnosis of hypertension and consuming antihypertensive medicines, or patients with a systolic blood pressure of 140 mm Hg, or a diastolic blood pressure of 90 mm Hg. Plasma levels of glucose, total cholesterol concentrations were measured by enzymatic colorimetric method (240 or Higher than 240 mg/dl).

After obtaining informed consent forms from all subjects, samples of peripheral blood were collected in EDTA-containing tubes. DNA was extracted using a Gene All kit. The ARMS-PCR method was employed for genotyping SNPs rs5882 and rs708272 RFLP-PCR and SNPs rs3917406, rs5355, rs17228212, and rs17465637.

### Genotyping by ARMS-PCR and RFLP-PCR methods

For genotyping SNPs rs3917406, rs5355, rs17228212, and rs17465637 by the ARMS-PCR method, three primers were made for each polymorphism. PCR was done in a 25-µl reaction volume comprising 2 µl of genomic DNA, 8 µl of ddH2O, 12 µl of Master Mix (Ampliqon, Odense, Denmark), and 1 µl of every specific primer (Table 1). Amplifying by PCR was started by initial denaturation at 95 °C (4 min), and then amplifying with 30 cycles of 95 °C (45 s), (rs3917406 [64 °C], rs5355 [66 °C], rs17228212 [58 °C], and rs17465637[59 °C] (45 s), 72 °C (45 s), finalized by extension at 72 °C (7 min). Then PCR product were subjected to electrophoresis on 2% agarose gel.

**Table 1:** Characteristics of primers (ARMS method)

Polymorphs	Primers Sequences 5**′** → 3**′**
rs3917406	F:5′-AAT AGC ATG GTT CAT GGA AAG TAC-3′ R1:5′-CTT TTC TCA TCT TTT CAA AAG TAA GAA-3′ R2: 5′-CTT TTC TCA TCT TTT CAA AAG TAA GAG-3′
rs5355	F:5′-TGT GGT GTG GTA TTG TGA ACG-3′ R1:5′-ATT TCC GAA GCC AGA GGA G-3′ R2: 5′-ATT TCC GAA GCC AGA GGA A-3′
rs17228212	F1:5′-TGT CTT TGC CGT CAT TGA TCT-3′ F2:5′-GTC TTT GCC GTC ATT GAT CC-3′ R: 5′-CCC GCT TGG ATC ATA ACT G-3′
rs17465637	F1:5′-AAA CCA TAA TAG TTA TGC TGA GAA GTT A-3′ F2:5′-AAC CAT AAT AGT TAT GCT GAG AAG TTC-3′ R: 5′-CTG TTC CGT GAC TGA AAA AAG-3′

The RFLP-PCR technique using one pair of the primers and one specific restriction enzyme was employed for genotyping SNPs rs5882 and rs708272 (Table 2). A 25-µl reaction volume containing 2 µl of genomic DNA, 8 µl of ddH2O, 12 µl of Master Mix (Ampliqon, Odense, Denmark), and 1 µl of every specific primer was prepared for PCR. The following settings were considered for PCR. Denaturation was initiated at 95 °C (4 min) and then amplification was done for 30 cycles consisting of denaturation at 95 °C (45 s), annealing (rs5882 [60 °C] and rs708272 [62 °C] for 45 s), extension at 72 °C (45 s), and an endpoint extension at 72 °C (7 min). The restriction enzyme was used to digest 10 μl of the PCR product at 37and 65 °C for Rsa1 and Taq1B, respectively, for about 12 h at night. The PCR fragments were subjected to electrophoresis on 2% agarose gel. The following are the final three genotypes of rs5882: GG (308 bps), AG (40, 268, and 308 bps), and AA (40, 268 bps), with GG (535bps), AG (174, 361, and 535bps), and AA (174, 361bps) as the three rs708272 genotypes. The Sanger sequencing technique was also applied to confirm the low number of results determined by ARMS-PCR and RFLP-PCR (available upon request).

**Table 2:** Characteristics of primers, restriction enzymes and products (restriction fragments)

Polymorphs	Primers Sequences 5**′** → 3**′**	Restriction enzyme	Products [bp]
rs5882	5′-GCA GAA CAG TAG TGG CCA AGC AGC G-3′ 5′-GCG GTG ATC ATT GAC TGC AGG AAG CTC TGT A-3′	Rsa1	A:40,268 G:308
rs708272	5′-CAC CCC AGA GAG AGG AGT GCC-3′ 5′-CTG AGC CCA GCC GCA CAC TAA C-3′	Taq1B	A:174, 361 G:535

### Statistics

Data were analyzed statistically using SPSS version 18.0. The different frequencies of alleles and genotypes between the case and control subjects were examined in terms of Hardy–Weinberg equilibrium by the Chi-square (X2) test. The relationship between the genotypes and CAD as well as between the clinical covariates (such as age, height, weight, and BMI) and CAD in the patient and healthy groups were compared using the independent samples test. A *p*-value of ≤ 0.05 was regarded as statistical significance. Moreover, the values for odds ratios (ORs) and 95% confidence intervals (95% CIs) were calculated to evaluate the relationship between the genotypes and the risk of CAD.

## Result

Table 3 summarizes the attributes of the CAD and non-CAD patients both clinically and biochemically. According to the test results, the control and patient subjects were statistically different concerning the height (*p*-value < 0.05), but they were almost similar in weight, age, and BMI (*p*-value > 0.05). As revealed by the results of the Chi-square test (× 2), the gender (OR = 2.36 (CI 1.36, 4.1); *p*-value = 0.002) and history of smoking (OR = 4.7(CI 1.0, 22.2); *p*-value = 0.035) were significantly different among control and patient groups, but no statistical differences were found for histories of hypercholesterolemia, diabetes, and hypertension (*p*-value > 0.05). The genotypes and allele frequencies of the *MIA3*, *SELE*, *SMAD3*, and *CETP*, polymorphisms are shown in Table 4. The TT genotype of the rs3917406 polymorphism was more frequent in the CAD patients than in the healthy group (*p*-value = 0.006). The CC genotype of the rs5355 polymorphism was more frequent in the CAD patients than in the healthy subjects (*p*-value = 0.022). A rising trend was noticed in the frequency of the GG genotype of the rs5882 polymorphism in male patients in comparison to that in healthy subjects (Table 5), which was different significantly (*p*-value = 0.001). The examined participants were not statistically different in terms of allele and genotype frequencies for the other polymorphisms, namely rs708272, rs17465637, and rs17228212.

**Table 3:** Clinical and biochemical characteristics of patients and controls

Parameters	Patient (n = 101)	Control (n = 111)	O.R(95% CI)	*p-*value
Age (years)	57.61 ± 11.44	57.24 ± 11.20		0.409
Height	166.91 ± 8.07	163.32 ± 10.64		0.0027**
Weight	77.64 ± 12.91	78.27 ± 11.14		0.356
BMI [kg/m^2^]	28.20 ± 4.88	29.85 ± 6.05		0.0143*
Sex, F/M	64/37	47/64	2.36 (1.36, 4.1)	0.002**
Hypercholesterolemia Yes/no	27/37	14/25	1.3 (0.57, 2.96)	0.527
Smoking Yes/no	14/46	2/31	4.7 (1.0, 22.2)	0.035*
Diabetes mellitus Yes/no	27/38	19/26	0.972 (0.45, 2.1)	0.943
Hypertension Yes/no	27/37	20/24	0.876 (0.404, 1.89)	0.737

*Differences statistically (*p* < 0.05)

**Differences statistically (*p* < 0.01)

**Table 4:** Genotype and allele frequencies in controls and patients

Polymorphisms	Genotypes			*p-*value	Alleles		O.R (95% CI)	*p*-value
*SELE* rs3917406 Controls (n = 111) Patients (n = 101)	CC (%) 43 (38.7%) 29 (28.7%)	CT (%) 48 (43.4%) 37 (36.6%)	TT (%) 20 (18%) 35 (34.7%)	0.02	C 134 (60.3%) 95 (47.0%)	T 88 (39.7%) 107 (53.0%)	0.58 (0.397, 0.857)	0.006
*SELE* rs5355 Controls (n = 111) Patients (n = 101)	CC (%) 87 (87.4%) 91 (90.15%)	CT (%) 22 (19.8%) 9 (8.9%)	TT (%) 2 (1.8%) 1 (1%)	0.043	C 196 (88.2%) 191 (94.6%)	T 26 (11.8%) 11 (5.4%)	2.3 (1.11, 4.79)	0.022
*CETP* rs5882 Controls (n = 100) Patients (n = 100)	GG (%) 28 (28%) 40 (40%)	GA (%) 52 (52%) 45 (45%)	AA (%) 20 (20%) 15 (15%)	0.195	G 108 (54%) 125 (62.5%)	A 92 (46%) 75 (37.5%)	1.4 (0.95, 2.12)	0.085
*CETP* rs708272 Controls (n = 100) Patients (n = 100)	GG (%) 36 (36%) 27 (27%)	GA (%) 43 (43%) 55 (55%)	AA (%) 21 (21%) 18 (18%)	0.217	G 115 (57.5%) 109 (54.5%)	A 85 (42.5%) 91 (45.5%)	0.885 (0.596, 1.31)	0.546
*MIA3* rs17465637 Controls (n = 80) Patients (n = 80)	AA (%) 61 (76.3%) 52 (65%)	AC (%) 18 (22.5%) 24 (30%)	CC (%) 1 (1.2%) 4 (5%)	0.19	A 140 (87.5%) 128 (80%)	C 20 (12.5%) 32 (20%)	0.57 (0.31, 1.05)	0.069
*SMAD3* rs17228212 Controls (n = 80) Patients (n = 80)	TT (%) 80 (100%) 80 (100%)	TC (%) 0 (0%) 0 (0%)	CC (%) 0 (0%) 0 (0%)	N.S	T 160 (100%) 160 (100%)	C 0 (0%) 0 (0%)	1	N.S

**Table 5:** Comparison Genotype and allele frequencies of rs5882 with risk of CAD in healthy and patient men and women

Polymorphism	Genotypes					Alleles		
	GG (%)	GA (%)	AA (%)	*p*-value	G	A	O.R (95% CI)	*p*-value
Men								
*CETP* rs5882							2.54 (1.44, 4.48)	
Controls (n = 42)	8 (19%)	21 (50%)	13 (30.9%)	0.007	37 (44.0%)	47 (56.0%)		0.001
Patients (n = 63)	30 (47.7%)	24 (38%)	9 (14.3%)		84 (66.6%)	42 (33.3%)		
Women								
*CETP* rs5882							0.79 (0.44, 1.42)	
Controls (n = 58)	20 (34.5%)	31 (53.5%)	7 (12%)	0.695	71 (61.2%)	45 (38.8%)		0.428
Patients (n = 37)	10 (27%)	21 (56.8%)	6 (16.2%)		41 (55.4%)	33 (44.6%)		

## Discussion

Cardiovascular diseases are one of the leading causes of death around the world. Coronary artery disease (CAD) is the most common heart disorder, caused by coronary artery stenosis and the formation of atheroma plaques [1]. Recent genome-wide association studies have found a link between elevated coronary artery disease susceptibility and particular SNPs within the genome that play a role in combination with other established CAD risk factors, but the exact mechanism remains unknown. Atherosclerosis is one of the key pathophysiological causes of CAD, according to clinical observation [31]. In the current research, the relationship of rs17465637 with susceptibility to CAD was examined among adults in Iran, and no significant association was detected between the rs17465637 in the *MIA3* gene and CAD (OR = 0.57(CI 0.31, 1.05); *p*-value = 0.069). In a meta-analysis, on the other hand, Xiuchun Li et al. presented evidence of a case–control association in the Chinese Han population with 2503 CAD patients and 2920 controls. Their report suggested a significant association of the SNP rs17465637 in *MIA3* with CAD (*p*-value = 0.01, OR = 1.11), with a rigorous confirmation by follow-up meta-analyses in five admixed Asian populations with 7263 CAD cases and 8347 controls for CAD. Highly significant relationships were reported between the SNP rs17465637 and CAD in the populations from Asia (*p*-value = 4.97 *10¯5, OR = 1.11). These data are strong supporters of the reality that the SNP rs17465637 in *MIA3* of populations in Asia confers a substantive risk of CAD [32]. Unlike our findings, GWAS mostly denotes the C allele of this variant as the risky one. The rs17465637-A, the rapid development of carotid plaques, and the elevated risk of cardiovascular incidents tended to be associated among rheumatoid arthritis patients with dyslipidemia, though, the logistic regression analysis showed that this was not significant [33]. From another point of view, rs17465637-C presented 20% greater odds (OR = 1.20; CI (1.12, 1.30)) of CAD in a European cohort examined in a meta-analysis of the WTCC and the German MI Family Study [3]; other meta-analyses represented the same findings [34–36]. Similarly, Han Chinese showed such a relationship, with (OR = 1.11; CI (1.02, 1.21)) [32]. Additionally, rs17465637-C showed associations with myocardial infarction among Japanese (OR = 1.45, *p*-value = 0.006) and white Americans (P-adjusted = 0.0034) [9, 37]. In a meta-analysis of 11 investigations from Europe, China, the United States, and New Zealand, 1q41 polymorphisms and CAD showed relationships and the C allele was the risky allele for CAD rather than the A allele [38]. However, allele A presented a protecting impact against CAD in a study by Wang et al. [9]. Saleem Ullah Shahid et al. found no statistical differences between the CAD cases and controls, hence, rs17465637 cannot be regarded as a risk factor for CAD independently in the examined population [39]. Atherosclerosis is characterized by a chronic inflammatory procedure, in which E-selectin is a mediator and its expression occurs on the vascular endothelium cell in response to several inflammatory stimuli [40]. The concentration of E-selectin rises in patients with atherosclerosis and CAD [41]. founded a relationship between the E-selectin polymorphism and E-selectin levels (*p*-value = 0.031) in the Taiwanese population was reported by Wu et al. [16]. Hongwei Shan et al. [42] also detected that the E-selectin rs3917406 polymorphism and CAD risk were significantly associated in a Han population of China. The authors discovered that the T allele of rs3917406 comprised 47.8% in the CAD group, which was statistically greater than that in the control group (42.8%, *p*-value = 0.009). In the present study, an association between the T allele of the rs3917406 polymorphism and the elevated risk of CAD was found in comparison to the C allele (OR = 0.58(CI 0.397, 0.857); *p*-value = 0.006). Regarding the next polymorphism rs5355, this research revealed that the E-selectin rs5355 SNP was significantly linked to CAD (OR = 2.3 (CI 1.11, 4.79); *p*-value = 0.022), which corresponds to findings in Mexican people [43]. As a result, it increases the risk of CAD by almost 2.5 times. Contrary to the current findings, Issac et al. investigated the Leu575Phe C1880T (rs5355) polymorphism and claimed that this SNP had no association with the risk of developing carotid atherosclerosis in end-stage renal disease in a population in Egypt [44]. Similarly, the research on the Han population revealed that the rs5355 C/T polymorphism rate was not significantly different in hypertension and control groups [45]. Therefore, the two SNPs in the *SELE* gene can differentially interact with other confounding factors and have relationships with the pathogenic activity of vascular disorders. The relationship of TGF-β1 and SMAD3 concentrations with the risk factors of CAD indicates the possible contribution of this cytokine to the formation of CAD by the regulation of atherogenesis. According to the present findings, the rs17228212 polymorphism has no association with the risk of CAD (*p*-value = 1.0), which corresponds to that of Saleem Ullah Shahid et al. who reported no connection between rs17228212 in the *SMAD3* gene and the risk of CAD [39]. Moreover, Vincent G Haver (2014) analyzed the link of seven SNPs, including rs17228212 at seven well-known CAD risk loci in 3,320 individuals with the diagnosis of systolic heart failure of ischemic etiology. All the seven loci showed no significant association with heart failure [46]. Samani et al. [3] on the contrary, identified several additional loci, namely 15q22.33 (rs17228212), which were significantly linked to CAD by a combinative analysis of both investigations. In susceptibility to CAD, the genetic variation of *CETP* mainly determines inter-individual variations. The contribution of the *CETP* rs708272 polymorphism to the recurring risk of CAD may be through the remodeling of anomalous HDL-C and by destroying the anti-atherogenic features of HDL-C. Therefore, it is possible to prevent and treat CAD by potentially targeting the genes involved in HDL. Our observations denote that the *CETP* rs708272 polymorphism is non-significantly associated with the risk of CAD in the Iranian population, which corresponds to those reported elsewhere indicating no association of the *CETP* rs708272 polymorphism with CAD [47, 48]. Contrary to the present findings, Bhanushali and Das [48], Rahimi et al. [49], Kaman et al. [50], and Iwanicka et al. [51] found positive relationships. Conflicting findings were observed in previous investigations on the impact of the next *CETP* rs5882 polymorphism on CAD [48, 52–54]. In a vast meta-analysis using 10,313 cases with coronary complications and 32,244 controls from 18 available investigations, rs5882 genotypes were associated with moderately inhibited CETP action (hence with the modest increase of HDL-C) and reversely with CAD [53]. Corresponding to this study, no relationship between the rs5882 polymorphism and the risk of coronary atherosclerosis has more recently been reported by some researchers [48, 54]. However, the association of rs5882 with CAD in healthy and diseased men groups was demonstrated by an additionally analyzed differential relationship (OR = 2.54(CI 1.44, 4.48); *p*-value = 0.001).

## Conclusions

In conclusion, the results of current case–control studies show the importance of polymorphisms.

genetic effects on risk of CAD in the Iranian population. According to our findings, it can be concluded that rs17228212, rs17465637, and rs708272 are not associated significantly with the risk of CAD. However, the rs5355 (*p*-value = 0.022) and rs3917406 (*p*-value = 0.006) in total subjects, and rs5882 (*p*-value = 0.001) in men patients were associated significantly with the risk of CAD. The different observations in our research and other investigations can arise from different sizes of surveyed subjects, race differences, and ignoring SNPs-SNPs and gene-environment interplays.

## Data Availability

The datasets generated and analyzed during the current study are available in the ClinVar. [SCV002515853–SCV002515858].

## Abbreviations

CAD: Coronary artery disease
MI: Myocardial infarction
MIA3: Melanoma inhibitory activity 3 gene
ABCA1: Adenosine triphosphate-dependent transporter to A-I apolipoproteins
LCAT: Lecithin-cholesterol-acyltransferase
RCT: Reverse cholesterol transfer
CETP: Cholesteryl ester transfer protein
SNP: Single nucleotide polymorphism
HDL-C: High-density lipoprotein cholesterol
